# Tuning of large piezoelectric response in nanosheet-buffered lead zirconate titanate films on glass substrates

**DOI:** 10.1038/s41598-017-00333-2

**Published:** 2017-03-21

**Authors:** Anuj Chopra, Muharrem Bayraktar, Maarten Nijland, Johan E. ten Elshof, Fred Bijkerk, Guus Rijnders

**Affiliations:** 1 Inorganic Materials Science Group, MESA+ Institute for Nanotechnology, University of Twente, PO Box 217, 7500AE Enschede, The Netherlands; 2 Laser Physics and Nonlinear Optics Group, MESA+ Institute for Nanotechnology, University of Twente, PO Box 217, 7500AE Enschede, The Netherlands; 3 Industrial Focus Group XUV Optics, MESA+ Institute for Nanotechnology, University of Twente, PO Box 217, 7500AE Enschede, The Netherlands

## Abstract

Renewed interest has been witnessed in utilizing the piezoelectric response of PbZr_0.52_Ti_0.48_O_3_ (PZT) films on glass substrates for applications such as adaptive optics. Accordingly, new methodologies are being explored to grow well-oriented PZT thin films to harvest a large piezoelectric response. However, thin film piezoelectric response is significantly reduced compared to intrinsic response due to substrate induced clamping, even when films are well-oriented. Here, a novel method is presented to grow preferentially (100)-oriented PZT films on glass substrates by utilizing crystalline nanosheets as seed layers. Furthermore, increasing the repetition frequency up to 20 Hz during pulsed laser deposition helps to tune the film microstructure to hierarchically ordered columns that leads to reduced clamping and enhanced piezoelectric response evidenced by transmission electron microscopy and analytical calculations. A large piezoelectric coefficient of 250 pm/V is observed in optimally tuned structure which is more than two times the highest reported piezoelectric response on glass. To confirm that the clamping compromises the piezoelectric response, denser films are deposited using a lower repetition frequency and a BiFeO_3_ buffer layer resulting in significantly reduced piezoelectric responses. This paper demonstrates a novel method for PZT integration on glass substrates without compromising the large piezoelectric response.

## Introduction

Perovskite oxides form a special and exciting class of materials which exhibit a wide spectrum of multifunctional physical properties such as superconductivity, photovoltaic effect, colossal magnetoresistance, ferroelectricity and piezoelectricity^1–5^. In particular, there has been an intensive research interest in using the piezoelectric response of ferroelectric thin films for a wide range of microelectromechanical systems (MEMS) such as in sensors and actuators^6–9^. Recently, an intriguing interest has been witnessed in integrating ferroelectric thin films on amorphous materials such as glass substrates for applications in data storage, electronic displays and adaptive optics^10–17^. Some of the electromechanical actuation based applications such as adaptive optics essentially demand high and stable piezoelectric response^17^.

The piezoelectric response in thin films is known to depend on composition, growth quality, orientation and size of the fabricated devices^7, 18–22^. Among all the ferroelectric materials Pb(Zr_x_Ti_1−x_)O_3_ with morphotropic phase boundary composition, Pb(Zr_0.52_Ti_0.48_)O_3_ (PZT), is usually preferred, not only for its high piezoelectric response, but also for the large remanent polarization and low coercive electric field^5, 9, 23, 24^. Therefore growth of well-oriented crystalline or epitaxial PZT films on glass substrates with control of the crystalline orientation is highly desired. Achieving epitaxial growth with (100) orientation is known to maximize the piezoelectric response compared to the polycrystalline films^18, 19^. In this regard, different strategies including growth of alternative buffers layers are being explored to manipulate the growth orientation. Epitaxial growth of oxide buffer layers such as Yttrium-stabilized zirconia (YSZ), CeO_2_ and SrTiO_3_ on natively oxidized Si substrates has been extensively reported in literature^25–27^. The presence of sub-nanometer thick (≥0.49 nm) oxide layer on Si substrates thermodynamically facilitates good quality crystalline growth of subsequent oxide layers by reducing/deoxidizing SiO_2_ completely and consequently promotes an epitaxial growth of oxide layers. However if the thickness of SiO_2_ layer on Si substrate is between 0.68 and 1.1 nm, a sub-nanometer thin layer of SiO_2_ was still observed under subsequent oxide layers in transmission electron microcopy measurements^28^. This SiO_2_, β-cristobalite, was found to be a crystalline phase of SiO_2_ which further assisted the epitaxial growth^25^. Furthermore, it has been reported that if the thickness of SiO_2_ on Si substrate exceeds 2 nm, the growth of subsequent oxide layers has been found to be completely polycrystalline due to inability of top oxide layer to deoxidize or assist the formation of crystalline SiO_2_^28^. Withal, direct deposition of LaNiO_3_ (LNO) films as a bottom electrode on glass substrates results in a polycrystalline growth with large resistivity values (~2.56 × 10^−5^ Ω.m) which hampers the direct integration of LNO on glass substrate^29^. Beside atomic layer deposited amorphous HfO_2_, which has been explored recently for the orientation controlled integration of PZT layers on Ni substrates^30^, use of crystalline nanosheets provides an alternative approach for the orientation controlled integration of PZT films on commercial substrates. Indeed, a seed layer of crystalline Ca_2_Nb_3_O_10_ (CNO) nanosheets has been reported to facilitate preferentially (001)-oriented LNO growth on glass substrate with reduced resistivity (~1.17 × 10^−5^ Ω.m)^29^. In spite of having numerous application possibilities, integration of ferroelectric films on commercial substrates still remain an apprehension due to high processing temperature. Crystallization utilizing laser assisted annealing is another alternative approach being developed to lower the processing temperature^31^. Laser annealing process involves a large absorption coefficient and short interaction time facilitating an efficient surface heating rather than volume heating and consequently being explored for the PZT integration on commercial substrates. Indeed, Tabata *et al.* ^32^ and Rajashekhar *et al.* ^33^ have used a second laser irradiation during pulsed laser ablation deposition to crystallize the PbTiO_3_ and PZT films, respectively.

However, the piezoelectric response is still drastically reduced compared to intrinsic piezoelectric response due to clamping of the thin film by the substrate^34–38^. On application of an electric field in the direction normal to the PZT film surface (longitudinal direction), the longitudinal expansion of the PZT film is coupled to a contraction in the direction parallel to its surface (transverse direction). Since the transverse contraction is constrained by the substrate, the effective longitudinal piezoelectric response of the PZT film is significantly reduced. The clamping effect is less pronounced for structures that have small lateral dimensions, thus a lot of research has been driven to fabricate island-like nanostructures with submicron lateral size to enhance the piezoelectric response^21, 39–41^. Indeed, experimentally it is probed that submicron size PZT capacitors have four times larger piezoelectric response in comparison to the large scale capacitors^40^. This larger piezoelectric response was later reported to be a primary manifestation of reduced clamping effect^41^. However, fabrication of island-like nanostructures and submicron size capacitors demand additional processing steps such as focused ion beam milling and chemical etching which increases the risk of contamination and damage. Recent reports^42, 43^ on growth of columnar arrays require depositions at elevated temperatures exceeding 700 °C therefore cannot be directly used for growth of PZT films on glass substrates that carry temperature sensitive amorphous structures^15^. Thus, the reduced piezoelectric response of films that have lateral sizes relevant for MEMS devices (up to hundreds of microns lateral size), where the clamping effect is most dominant, still remains as a challenge.

Here we report on hierarchically ordered columnar growth of crystalline PZT films with preferred (100) orientation on glass substrates at a maximum processing temperature of 600 °C and minimization of the clamping effect by tuning the growth conditions. Locally epitaxial growth and control on growth orientation was achieved by utilizing crystalline nanosheets of Ca_2_Nb_3_O_10_ (CNO) as seed layers on the glass substrates as explained in detail with relevant literature^29^ in our previous report^44^. LaNiO_3_ (LNO) electrodes and the PZT films were grown on the nanosheet-buffered glass substrates using pulsed laser deposition (PLD). PLD is a proven technique to grow high quality multifunctional oxide thin films and allows precise control on the growth quality by tuning of the deposition parameters such as laser fluence, repetition frequency of laser pulses, growth temperature and background gas pressure^45–48^. Most important for this work is the ability to change the microstructure of a film by changing the repetition frequency of the laser pulses. The following three heterostructures, named H_20Hz_, H_5Hz_ and H_BFO_ hereafter, were deposited with different repetition frequency of the laser pulses. Within H_BFO_, a BiFeO_3_ (BFO) buffer layer is used.

$${{\rm{H}}}_{20{\rm{Hz}}}:\mathrm{LNO}\,(5\,\,{\rm{Hz}})/{\bf{PZT}}\,({\bf{5}}\,{\bf{and}}\,{\bf{20}}\,{\bf{Hz}})/\mathrm{LNO}\,(5\,{\rm{Hz}})/\mathrm{CNO}/\mathrm{Glass},$$

$${{\rm{H}}}_{5{\rm{Hz}}}:\mathrm{LNO}\,(5\,{\rm{Hz}})/{\bf{PZT}}\,({\bf{5}}\,{\bf{Hz}})/\mathrm{LNO}\,(5\,{\rm{Hz}})/\mathrm{CNO}/\mathrm{Glass},$$

$${{\rm{H}}}_{{\rm{BFO}}}:\mathrm{LNO}\,(5\,{\bf{Hz}})/{\bf{PZT}}\,({\bf{5}}\,{\bf{Hz}})/{\bf{BiFe}}{{\bf{O}}}_{{\bf{3}}}\,({\bf{5}}\,{\bf{Hz}})/\mathrm{LNO}\,(5\,{\rm{Hz}})/\mathrm{CNO}/\mathrm{Glass}.$$

## Results and Discussion

**Figure 1:**
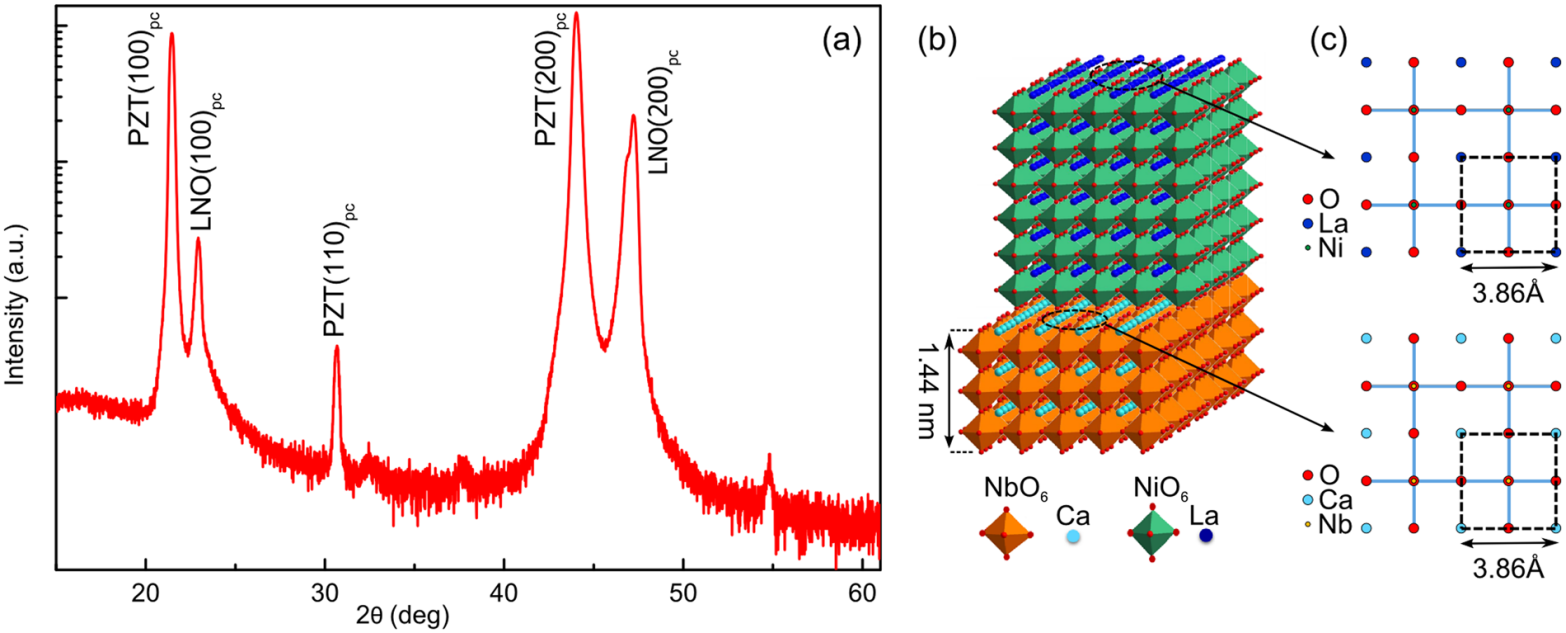
Crystal structure of the H_20Hz_ heterostructure. (**a**) XRD *θ–2θ* scan. (**b**) Polyhedral representation of LaNiO_3_ perovskite deposited on a perovskite-related Ca_2_Nb_3_O_10_ nanosheet. (**c**) The square in-plane lattice of the LaNiO_3_ and Ca_2_Nb_3_O_10_. The ideal fitting of the lattice parameters resulting in (100)_pc_ growth (subscript “pc” stands for pseudo-cubic indexing).

**Figure 2:**
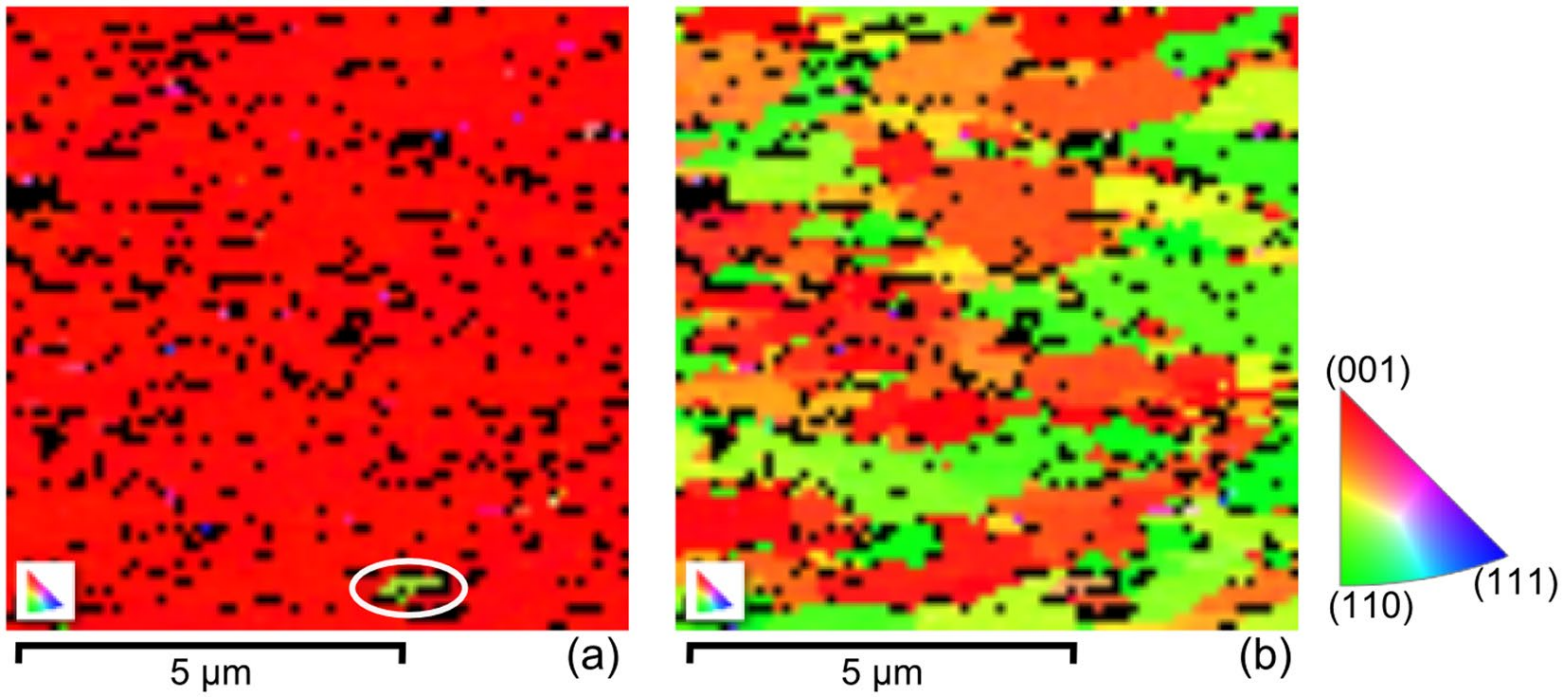
Pole figure maps in the (**a**) out-of-plane and (**b**) in-plane of the PZT film generated using electron backscatter diffraction measurements.

The XRD patterns reveal a predominantly (100)_pc_ oriented PZT growth as shown in Fig. 1(a) (shown only for H_20Hz_). The subscript “pc” stands for the pseudo-cubic indexing which is used for all the materials in this paper. However a minor (110)_pc_ reflection, which is two orders of magnitude smaller than the (100)_pc_ peak, is also observed. The PZT films for all three heterostructures were found to have a pure perovskite phase without the presence of any impurity or pyrochlore phase. Only (100)_pc_ orientation was observed for the LNO electrodes. It is also worth noting here that PZT films were also found in purely (100) orientation up to 750 nm^44^. The (100)_pc_ oriented growth of the LNO bottom electrode is facilitated by the match of its lattice parameters to the underlying CNO nanosheets as schematically illustrated in Fig. 1(b) and (c). The CNO nanosheets are known to have a 2D square lattice with a lattice parameter of *a*_CNO_ = 3.86 Å which matches the in-plane lattice parameter of the LNO pseudo-cubic unit-cell as shown schematically in Fig. 1(c) ^49, 50^. In this study, the lateral size of the CNO nanosheets deposited on glass substrates were around 2 µm^50^. Pure (100)_pc_ oriented growth of the LNO bottom electrode facilitates a (100)_pc_ oriented growth of the subsequent PZT layer. In addition to the XRD characterization, in-plane and out-of-plane crystal orientation of the PZT film for H_20Hz_ was mapped using EBSD technique. The generated out-of-plane and in-plane pole figure maps are shown in Fig. 2(a) and (b), respectively. The out-of-plane pole figure map in Fig. 2(a) demonstrates a dominant (100)_pc_ orientation (above 99% of the measured area) as almost the whole map has a single color (red color representing (100) orientation). A minor amount of (110)_pc_ orientation (encircled region that has green color representing (110) orientation) is also observed, which is indeed in accordance to our XRD observations. The in-plane pole figure map in Fig. 2(b) consists of predominately two colors representing the (100)_pc_ and (110)_pc_ orientations which signifies that the PZT film is randomly oriented in-plane. This in-plane random orientation of the PZT film is a manifestation of the random in-plane orientation of the CNO nanosheets lying underneath.

**Figure 3:**
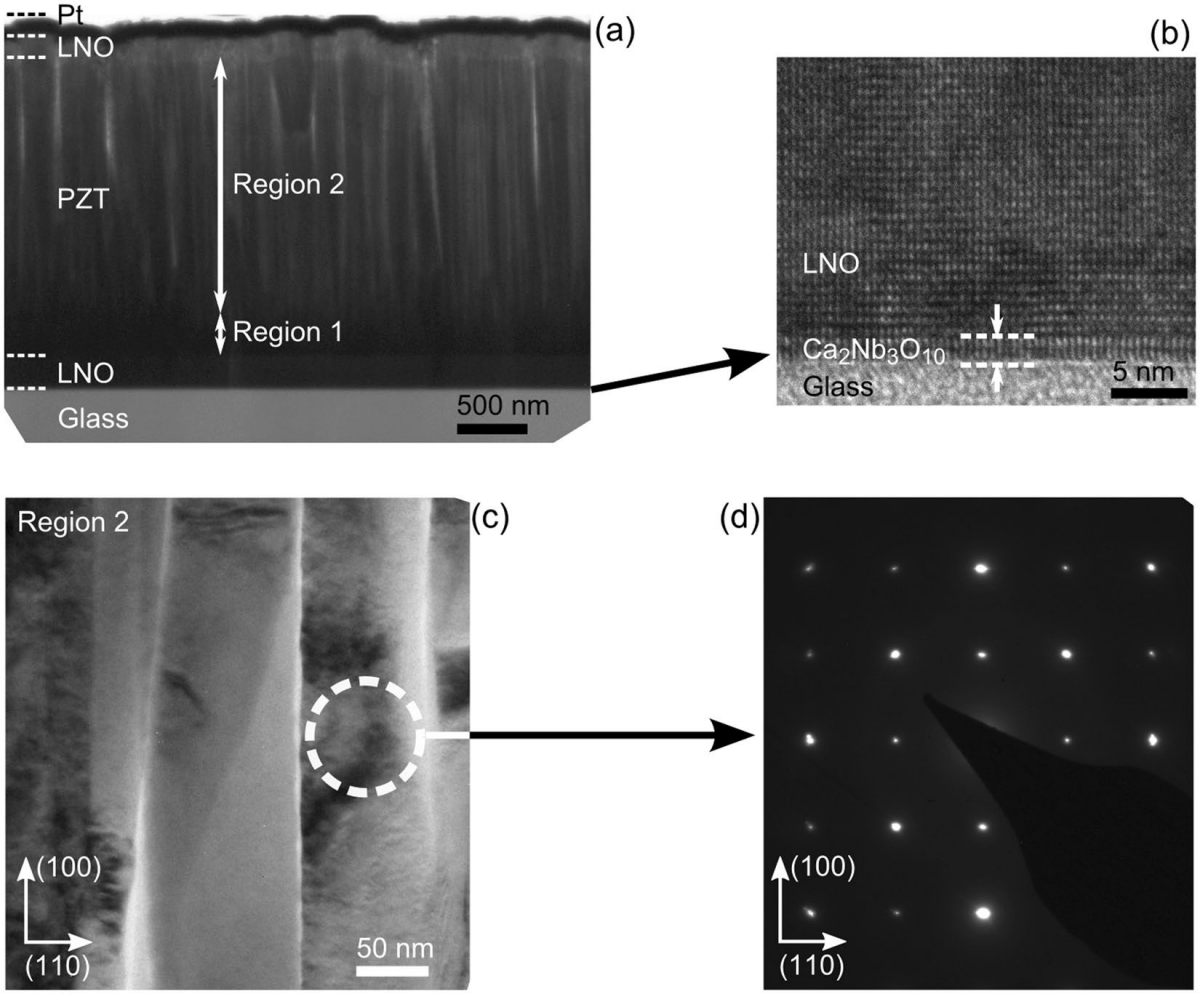
TEM images. (**a**) Cross-section of the H_20Hz_ heterostructure in which PZT layer was first deposited at 5 Hz repetition frequency (region 1 with dense packing) and then 20 Hz repetition frequency (region 2 with separated columns). (**b**) A magnified image of Ca_2_Nb_3_O_10_ nanosheet and glass interface. (**c**) A magnified image of the columns from region 2 of the PZT film. (**d**) Selected area electron diffraction pattern recorded for one of the column confirming an epitaxial growth.

Detailed microstructure and thickness investigations were performed using transmission electron microscopy (TEM). A cross-sectional TEM image of a 2 µm thick PZT film on a 200 nm thick LNO bottom electrode for the H_20Hz_ heterostructure is shown in Fig. 3(a). The thicknesses of both top LNO and Pt electrodes were found to be ~100 nm. A magnified image was captured in order to analyze the interface quality between the glass substrate and the nanosheets as shown in Fig. 3(b). A sharp and abrupt interface is visible between the glass and nanosheets, which confirms the effectiveness of using nanosheets as seed layers to grow a high quality crystalline LNO layer on an amorphous glass substrate. Further analysis of the microstructure for H_20Hz_ heterostructure reveals that the PZT film can be distinctively identified into two regions along the growth direction as: (a) region 1 at the bottom where the PZT film has a dense structure, (b) region 2 at the top where the PZT film grows into separated columns. This difference in the microstructure of the PZT film was controlled by changing the frequency of the laser repetition. As discussed in the experimental section, first 250 nm of the PZT film was grown with a frequency of 5 Hz laser repetition which resulted in a densely packed growth as seen in region 1. After ensuring the full coverage of LNO bottom electrode with 5 Hz repetition frequency, the frequency was increased to 20 Hz which resulted in films with separated columnar structures as shown in region 2. On magnifying the separated columnar region (region 2) as shown in Fig. 3(c), distinct columns with lateral sizes around 100 nm can be seen. The crystallography of these columns was analyzed using selected area electron diffraction (SAED). The SAED pattern for one of the columns that is shown in Fig. 3(d) confirms an epitaxial growth.

**Figure 4:**
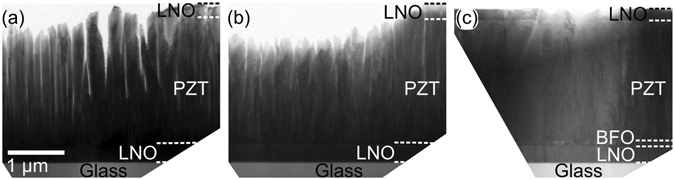
Cross-sectional TEM images revealing the impact of growth conditions on the heterostructures (**a**) H_20Hz_, (**b**) H_5Hz_, and (**c**) H_BFO_.

For a clear comparison of the microstructures, cross-sectional TEM images of the three heterostructures are shown in Fig. 4. The heterostructure H_20Hz_ shown in Fig. 4(a) has narrower (especially at the left half of the image) and more separated columns compared to other heterostructures. More importantly, the voids in H_20Hz_ are penetrating much deeper into the PZT film compared to H_5Hz_ shown in Fig. 4(b). The heterostructure H_5Hz_ still has a columnar microstructure in the upper half, but a non-columnar and continuous microstructure is visible in Fig. 4(c) for H_BFO_. The mechanism behind the transition from smaller and separated columnar growth in H_20Hz_ to a wider and more connected columnar growth in H_5Hz_ can be explained by considering the formation of islands during PLD. Guan *et al.* studied the effect of repetition frequency on the island size and island density using kinetic Monte-Carlo method and concluded that higher repetition frequency leads to formation of more and smaller islands as in our observation^51^. On the other hand, when the repetition frequency is lower, the islands have more time to become larger in lateral size. Similar results have been predicted and experimentally reported for BaTiO_3_ films grown using PLD^52, 53^. The transition from the partly columnar growth in H_5Hz_ to continuous growth in H_BFO_ can be explained by considering the lattice mismatch between the layers. The role of lattice-mismatch in controlling the quality of the films is also well studied^45, 46, 48^. The lattice parameters for the pseudo-cubic unit cells of LNO and PZT layers are *a*_LNO,pc_ = 3.86 Å and *a*_PZT,pc_ = 4.06 Å, respectively. The lattice mismatch between the PZT and LNO is 5.18% [=(*a*_PZT,pc_ − *a*_LNO,pc_)/*a*_LNO,pc_ × 100] which results in a columnar growth as observed for PZT in heterostructures H_20Hz_ and H_5Hz_. In order to further increase the density of the structure, a 50 nm thick BFO layer was used as a buffer layer between the LNO and PZT films. The pseudo-cubic unit cell of BFO has a lattice parameter of *a*_BFO,pc_ = 3.96 Å which reduces the lattice mismatch from 5.18% (between PZT and LNO) to 2.52% (between PZT and BFO) and hence promotes a denser PZT growth as evident from Fig. 4(c). All in all, the TEM images in Fig. 4 demonstrate that the columnar structure of the PZT film is dramatically influenced by the deposition parameters and can be tuned either by controlling the repetition frequency of the laser pulses or by using suitable buffer layers.

**Figure 5:**
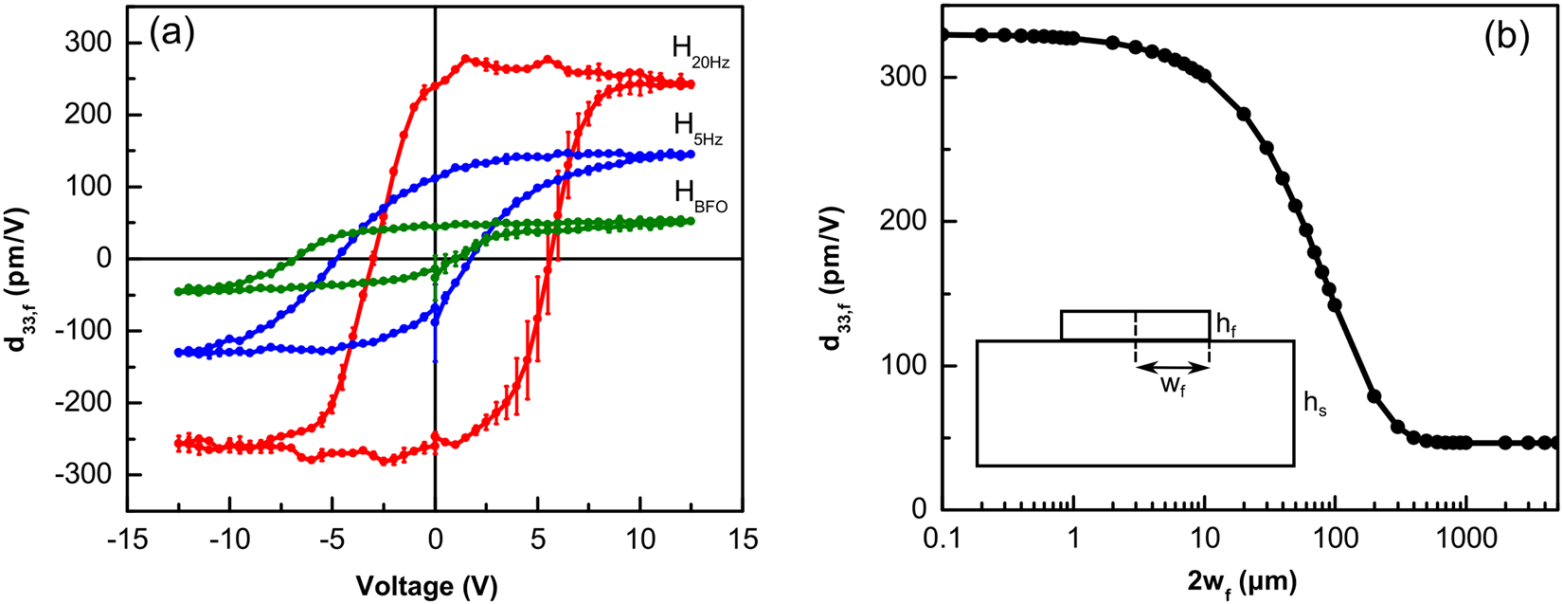
Piezoelectric response. (**a**) Longitudinal piezoelectric response (*d*_33,f_) of the three heterostructures measured using a laser Doppler vibrometer. Error bars represent the standard deviation. (**b**) Reduction of the piezoelectric response with increasing island width.

The measured longitudinal piezoelectric responses of the PZT films (*d*_33,f_) are shown in Fig. 5(a). A large effective piezoelectric coefficient of maximum 280 ± 2.88 pm/V (remanent piezoelectric coefficient is 250 ± 6.27 pm/V) was observed for the H_20Hz_ heterostructure that has separated columns. However, much lower piezoelectric coefficients of maximum 140 ± 1.90 and 50 ± 3.1 pm/V (remanent piezoelectric coefficients are 90 ± 19.1 and 30 ± 12.1 pm/V) were measured for the H_5Hz_ and H_BFO_ heterostructures with denser microstructures, respectively. This large decrease in the effective piezoelectric response can be attributed to the increase in the clamping effect with increasing lateral size of the islands. The piezoelectric response in a thin film can be related to the intrinsic piezoelectric response (*d*_33_) by^21, 22, 34, 35^:

1 $${d}_{33,f}={d}_{33}-\frac{2{s}_{13,f}{\sigma }_{f}}{{E}_{3}}$$

where *s*_13,f_ is the compliance of the film, *σ*_f_ is the in-plane stress of the film that is causing the clamping and *E*_3_ is the electric field applied in the out-of-plane direction. Considering that *d*_33_, *s*_13,f_ and *E*_3_ are independent of the island size, *σ*_f_ is the important parameter determining the dependence of *d*_33,f_ to the island size. To explain better the in-plane stress, we consider the simplified geometry of the film and substrate shown in Fig. 5(b). In this geometry, *w*_f_, *h*_f_ and *h*_s_ are the half-width of the film island, thickness of the film and thickness of the substrate, respectively. An approximate formula of the in-plane stress as a function of *x* (distance to the center of the island) has been derived by Suhir as^54^:

2 $${\sigma }_{f}(x)={Y}_{f}^{0}\chi (x){\varepsilon }_{f}.$$

The parameter ${Y}_{f}^{0}$ is the generalized Young’s modulus^54^ or the biaxial modulus^55^ of the film that commonly replaces the Young’s modulus (*Y*_f_) to account for the two-dimensional stresses in thin film geometries. In our case, the film is anisotropic and has a columnar texture with columns aligned in the out-of-plane direction. For such films the generalized Young’s modulus can be written as^56^:

3 $${Y}_{f}^{0}=\frac{1}{{s}_{11,f}+{s}_{12,f}}.$$

The function *χ*(*x*) characterizes the distribution of the stress along the film width that is given by^54^ :

4 $$\chi (x)=1-{e}^{-k({w}_{f}-x)}\,{\rm{with}}\,k=\sqrt{\frac{3}{2}(\frac{{s}_{11,f}+{s}_{12,f}}{{h}_{f}})\frac{{Y}_{s}}{{h}_{s}(1+{\nu }_{s})}}$$

where *Y*_s_ and *v*_s_ are the Young’s modulus and Poisson’s ratio of the substrate, respectively. The parameter *ε*_f_ is the in-plane strain of the film defined as *ε*_f_ = *d*_31_ *E*_3_. To demonstrate the dependence of the effective piezoelectric response to the island width with a relevant example, we consider the theoretical elastic and intrinsic piezoelectric parameters of the tetragonal PbZr_0.5_Ti_0.5_O_3_ material that has a composition very close to our films. The effective piezoelectric response (*d*_33,f_) is calculated using the parameters^18, 57, 58^ in Table 1 and plotted in Fig. 5(b). For convenience, island half-width is replaced with island width in the figure. It is clearly visible from the figure that the effective piezoelectric response decreases from the intrinsic piezoelectric value to a lower, *i.e.* clamped, piezoelectric value as the island width increases. The effective piezoelectric response for an island width close to our column width (~100 nm) is 330 pm/V that is close to our measured piezoelectric response of 250 ± 6.27 pm/V. The difference between the calculated and measured value is due to the clamped part of the columns at the bottom of the film (region 1 in Fig. 3). The calculated piezoelectric response for the island width matching our continuous film width (200 µm) is 78 pm/V that is again close to our measured piezoelectric response of 30 ± 12.1 pm/V. The reduced piezoelectric response of our films compared to calculated value can be due to additional clamping from the sides of the film which can cause effectively a larger island width and was not taken into account in the calculation. Lastly, the reason for the relatively smaller piezoelectric response of the H_5Hz_ heterostructure (90 ± 19.1 pm/V) can be explained by considering its microstructure. The separated columns in H_5Hz_ heterostructure are visible only in the upper half of the film, which results in a significant reduction of the piezoelectric response due to the contribution from the lower continuous part. Here, we also remark that the effective piezoelectric coefficient of maximum 280 pm/V is, to the best of our knowledge, the highest piezoelectric coefficient measured on glass substrates^44, 59^.

**Table 1:** Thickness, elastic and piezoelectric parameters of the film and the substrate used in the calculations.

Material	*H* [µm]	*Y* [GPa]	*v*	*s*_11_ [1/TPa]	*s*_12_ [1/TPa]	*s*_13_ [1/TPa]	*d*_31_[pm/V]	*d*_33_ [pm/V]
PbZr_0.5_Ti_0.5_O_3_^[^^18, 57^^]^	2	—	—	5.76	−0.28	−4.98	−156	330
Glass substrate^[^^58^^]^	500	67.6	0.17	—	—	—	—	—

Residual stress is an important aspect that should be considered in thin films developed for applications requiring surface flatness such as adaptive optics. In general, residual stress is composed of contributions from intrinsic stress and thermal stress. Intrinsic stress can arise due to lattice mismatch and phase change during cooling, and can be released especially in thick films through orientation change and structural changes such as domain formation^60^. Thermal stress builds up during cooling of the sample from the deposition temperature to room temperature due to difference in the thermal expansion coefficients of the film and the substrate. The reported residual stress values for PZT and LNO films are in the range of 113–189 MPa^60–63^ and 60 MPa^63^, respectively. This level of stress can be managed through depositing buffer layers counteracting the residual stress or depositing a symmetric version of the layers at the back side of the substrate^64^. Furthermore it is expected that open columnar microstrucure presented in this paper can help to release the residual stress. Nevertheless, a detailed investigation of the residual stress requires an extensive study that reaches beyond the scope of this paper and will be carried out in the future.

**Figure 6:**
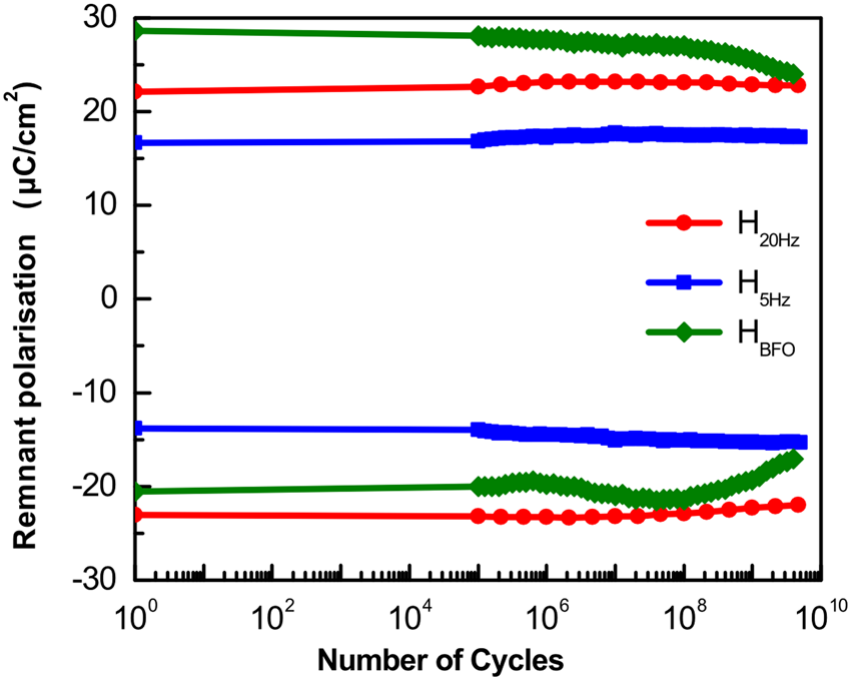
Plot of remanent polarization versus number of switching cycles.

Last but not the least, for device applications of these films, a long-term switching stability on applying external electric field is essential. The stable operation of PZT films for all the three heterostructures was confirmed using fatigue measurements as shown in Fig. 6. Up to the tested 5 × 10^9^ operating cycles, the remanent polarization of the heterostructures H_20Hz_ and H_5Hz_ are stable, which demonstrate the applicability of these films in device applications for future technology. For the heterostructure H_BFO_ a decrease in the remanent polarization is observed after 10^8^ cycles possibly due to BFO interface.

## Conclusion

In summary, we demonstrated a novel approach to tune the piezoelectric response of PZT films by controlling the microstructure of the hierarchically ordered columns, without any chemical treatment. The microstructure was controlled either by changing the repetition frequency of PLD process or by introducing a suitable BFO buffer layer. PZT films deposited with 20 Hz repetition frequency showed separated columns which manifested a large piezoelectric response of 250 pm/V due to reduced substrate induced clamping effect. In case of films deposited with 5 Hz repetition frequency either directly on LNO or on BFO buffered samples, densely packed columnar or continuous growth resulted in a reduced piezoelectric response which is conclusively demonstrated the effect of the substrate clamping. To conclude, this work offers new possibilities to tune the piezoelectric response without any chemical treatment, which opens new avenues in thin film fabrication for the future device applications. The same approach can be extended to other oxide systems as well to tune their response.

## Experimental methods

### Fabrication

In order to promote crystalline growth and control the growth orientation of the subsequent layers, CNO nanosheets were deposited on glass substrates. The CNO nanosheets were exfoliated by chemical processing from their layer-structure parent compound KCa_2_Nb_3_O_10_. In this process, the parent compound material KCa_2_Nb_3_O_10_ was first treated with nitric acid to obtain a protonated compound which was then exfoliated to CNO nanosheets on further treatment with exfoliation agent tetrabutylammonium hydroxide. The exfoliated nanosheets were transferred to the glass substrates using Langmuir-Blodgett deposition process at room temperature. The glass substrates used in this article were of 500 μm thickness. Prior to the transfer of nanosheets, the glass substrates were first cleaned on a hot plate at 250 °C with a jet of supercritical CO_2_ followed by oxygen plasma cleaning. More experimental details of the nanosheet preparation and deposition on glass substrates can be found elsewhere^49^.

The nanosheet coated glass substrate was loaded into the PLD chamber and a base pressure of 5 × 10^−7^ mbar was maintained before raising the substrate temperature. The nanosheet coated substrate was annealed at 600 °C for 60 minutes in 0.14 mbar oxygen pressure. All the oxide layers on the nanosheet coated glass substrates were deposited *in situ* by ablating materials from their respective stoichiometric targets using PLD with a KrF excimer laser operating at 248 nm wavelength with a pulse duration of 20 ns. Electrical measurements were facilitated using the LNO bottom and top electrodes. The LNO electrodes were deposited at 600 °C with a laser fluence and repetition frequency of 1.5 J/cm^2^ and 5 Hz, respectively. For the PZT layer, PbZr_0.52_Ti_0.48_O_3_ morphotropic phase boundary (MPB) composition was used to harvest the highest piezoelectric response. PZT films were deposited at 585 °C in 0.27 mbar oxygen pressure with a laser fluence of 2 J/cm^2^. During deposition of the PZT layer for the first heterostructure (H_20Hz_), a dense PZT seed layer (~250 nm) was deposited with a frequency of 5 Hz repetition to avoid the short circuiting between the top and bottom electrodes. Then the frequency of laser repetition was switched to 20 Hz for the rest of the deposition. The PZT films were deposited completely with a frequency of 5 Hz laser repetition for both H_5Hz_ and H_BFO_ heterostructures. A BiFeO_3_ (BFO) buffer layer was deposited with the same growth conditions as LNO layers for H_BFO_ heterostructure. A 100 nm thick platinum (Pt) layer was deposited by radio frequency sputter deposition at room temperature on the top LNO electrode for all the heterostructures. The Pt layer deposited at the top improves the homogeneity of the electric field across the top electrode. Top electrodes with 200 × 200 µm^2^ area were patterned using a standard photolithography process and structured by dry argon etching.

### Characterization

The crystallographic properties of the samples were analyzed using an x-ray diffractometer (Philips X’Pert MRD) with Cu-*Kα* radiation. Pole figure maps were generated from electron back scattering diffraction (EBSD) patterns of the PZT films recorded using a high-resolution scanning electron microscope (HR-SEM, Zeiss MERLIN). The microstructure of the samples was analyzed by using transmission electron microscopy (TEM, Philips CM300ST - FEG operating at 300 kV). Mechanical and ion-beam etching based techniques were employed for sample preparation for TEM analysis. Fatigue measurements were recorded using a ferroelectric tester (TF analyzer 2000, aixACCT). The fatigue measurements were performed using fatigue pulses of 15 V amplitude with a frequency of 100 kHz. After each fatigue cycle, the *P-E* hysteresis loop was measured with a triangular pulse of 30 V amplitude at 1 kHz frequency. The macroscopic piezoelectric responses of the films were measured using a laser Doppler vibrometer (LDV, Polytec MSA-400) operating at 8 kHz. To carry out the LDV measurements, all the samples were glued to a large metal plate with silver paste to impede the bending of the substrates. The LDV measurements were performed by applying an AC signal with a sub-coercive field peak-to-peak amplitude of 0.5 V superimposed on a DC voltage sweeping from −12.5 V to +12.5 V. LDV was also used in scanning mode to record line profiles across the top of electrodes. Line scans demonstrate that the deflection is zero on the top surface immediately outside an exited electrode^17^. This observation and gluing the samples assure that the measured piezoelectric response is solely due to effective out-of-plane piezoelectric coefficient.
